# Scar sarcoidosis on a finger mimicking a rapidly growing soft tissue tumour: a case report

**DOI:** 10.1186/1756-0500-5-545

**Published:** 2012-10-02

**Authors:** Marcel-Philipp Henrichs, Arne Streitbürger, Georg Gosheger, Carsten Surke, Christian Dierkes, Jendrik Hardes

**Affiliations:** 1 Department of General Orthopaedics and Tumourorthopaedics, University Hospital Münster, Münster, Germany; 2 Department of Traumatology, Reconstructive- and Handsurgery, University Hospital Münster, Münster, Germany; 3 Centre for Histology, Cytology and Molecular Diagnostics, Trier, Germany

**Keywords:** Scar sarcoidosis, Index finger, Soft tissue sarcoma, Systemic therapy

## Abstract

**Background:**

Scar sarcoidosis is a rare and uncommon but specific cutaneous manifestation of sarcoidosis. In general it arises in pre-existing scars deriving from mechanical traumas. As most surgeons dealing with scars might not be aware of cutaneous sarcoidosis and its different types of appearance the appropriate staging and treatment might be missed or at least delayed. To our knowledge this is the first case in literature of scar sarcoidosis on a finger.

**Case presentation:**

We present a case of a 33-year-old carpenter who developed scar sarcoidosis on his right index finger 4 years after the tendon of the long digital flexor got accidentally cut by an angle grinder. He was referred due to a swelling of the finger suspected to be a malignant soft tissue tumour. The circumference of the affected finger had almost doubled, adding up to 94 mm. Incision biopsy revealed typical noncaseating granulomas. Further investigation showed a systemic extent of the disease with involvement of the lung. A systemic treatment with oral steroids led to an almost full regression of the swelling with restoration of function and resolution of lung infiltrates.

**Conclusion:**

In case of a suspicious and/or progressive swelling a definite diagnosis should be achieved by biopsy within a short time to enable a proper treatment. If scar sarcoidosis is proven further investigation is necessary to exclude a systemical involvement. A surgical treatment of the swelling is not indicated.

## Background

Sarcoidosis is a systemic inflammatory disorder with formation of noncaseating granulomas. The incidence of this multisystem disease is 10–15 cases per 100.000 per year. Broad American studies show that sarcoidosis is 10–17 times more common in African American than in Caucasians [1]. In most cases lymphadenopathy can be observed, other organs like lung and liver are also often involved. In 9% to 37% a cutaneous involvement can be observed [2-6]. Scar sarcoidosis is a rare but specific manifestation of cutaneous sarcoidosis, occuring in 2.9% - 29% of overall cases [2,4-6]. It arises in the area of older scars after mechanical harm of the skin and can lead to a loss of function of the affected area [3,7]. Information regarding the appearance of the lesions differs. In most cases an evident inflammation with a red-purpulish aspect and induration is described [2]. Important differential diagnoses like a sarcoma or a keloid should be considered. Incision biopsy is needed to provide a diagnosis. In case of scar sarcoidosis further investigation on systemic involvement have to be performed. We present the first case of scar sarcoidosis of the finger.

## Case presentation

**Figure 1:**
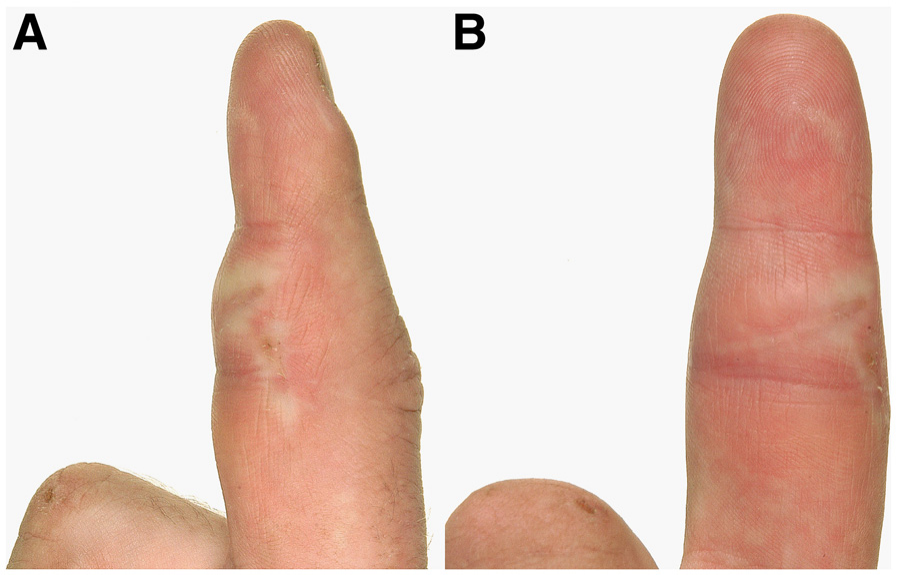
**A and B: lateral (A) and palmar (B) view of the right index finger: palmar swelling in the area of the base and middle phalanx after 4 weeks of corticosteroid therapy.** Compared to the initial finding the swelling is already diminished. On both pictures the indurated scar is prominent.

We report on a 33-year-old carpenter with a solid swelling in the area of the palmar base and middle phalanx of his right index finger (Figure 1A and B). He was referred due to suspicion of a malign tumour 8 weeks after he noticed the continuously growing non-aching swelling. At date of presentation he could hardly flex the finger in the proximal interphalangeal joint, range of motion was restricted to extension/flexion 0/0/10 degrees (according to Neutral Zero Method). The range of motion in the metacarpophalangeal and distal interphalangeal joint was also limited. Compared to the left side the circumference of the affected finger had almost doubled to 94 mm. Further, we detected a small scar on the palmar side in the area of the maximal swelling.

The patient told us that this scar derived from an injury 4 years ago when the tendon of the deep digital flexor was cut by an angle grinder. After suturing the tendon appearance and function of the finger had been fully restored.

**Figure 2:**
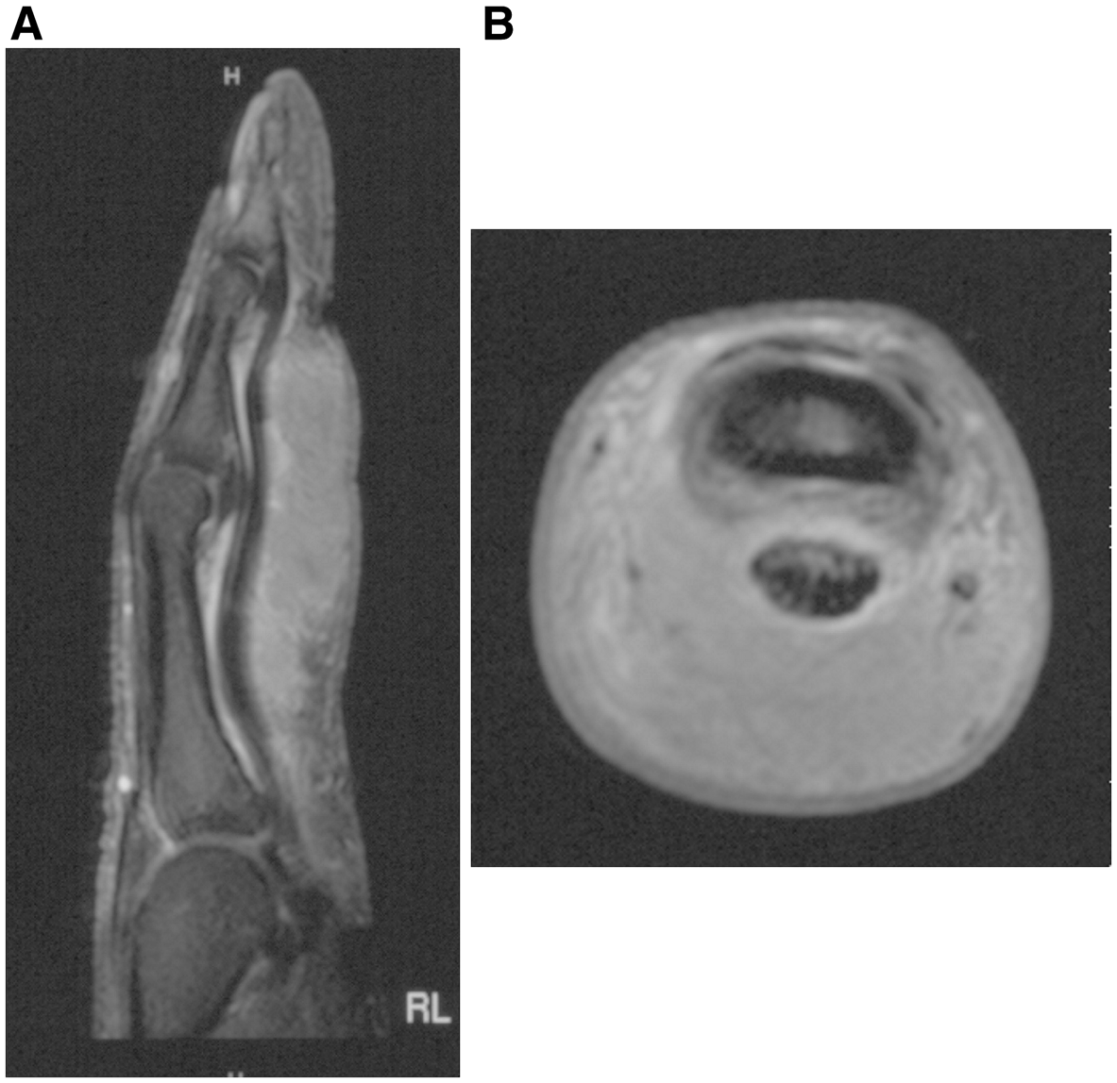
**A and B: MRI scans of the right index finger, sagittal (A) and axial (B) slide, T1 with contrast medium.** The slides show the size of the swelling based on the long flexor tendon with extension into the dermis. The mass is almost homogenious with a slight enhancement of contrast medium around the tendon. No change in bony structures.

Apart from enlarged soft tissue X-rays of the finger were unsuspicious. MRI-scans showed a considerable palmar soft tissue swelling with an enhancement of contrast medium with contact to the flexor tendon (Figure 2A and B). Blood tests including complete blood count and the inflammation markers C-reactive protein and erythrocyte sedimentation rate remained without pathological findings.

**Figure 3:**
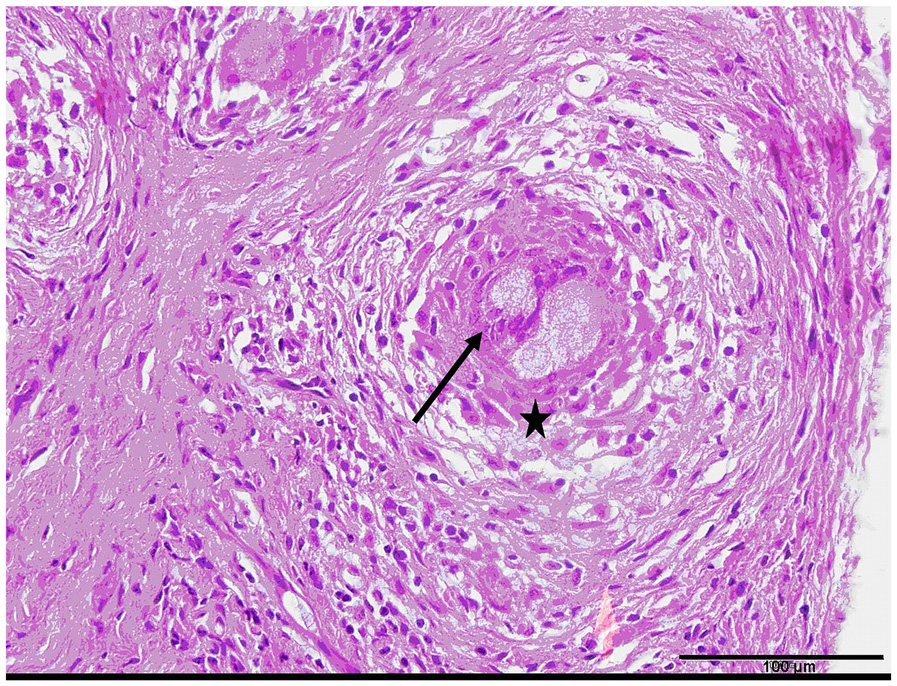
**Hematoxylin and eosin (HE) staining, 40-fold magnification (bar indicating 100 μm): histological slide of the biopsy specimen.** The picture shows a typical noncaseating granuloma consisting of Langhans’ multi-nucleated giant cells (arrow) and Epithelioid cells (*), surrounded by leucocytes.

**Figure 4:**
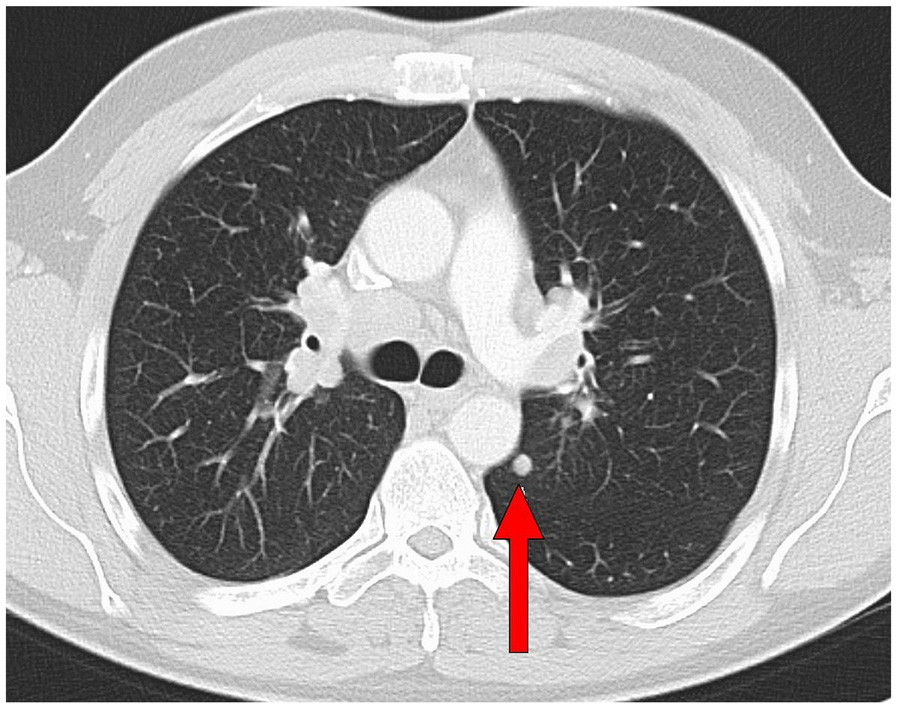
**axial slide of a CT-scan of the chest, lung window.** Findings before the start of the systemic treatment. Hilar lymphnodes are swollen, the arrow indicates a nodal infiltrate of the left lung.

For diagnosis and exclusion of the existence of a malignant and/or local aggressive soft tissue tumour we performed a small incision biopsy. The result of the histopathological examination was scar sarcoidosis with detection of typical noncaseating granulomas (Figure 3). The patient was referred to the department of internal medicine for further diagnostic investigation and therapy. CT-scans of his chest revealed hilar lymphnode swelling as well as nodal and lamellar infiltrates in both lungs consistent with stage II radiographic sarcoidosis (Figure 4). Nevertheless, the initial vital capacity of the lung was within normal range (7.11 liter). The initial angiotensin converting enzyme (ACE) level was only slightly increased (59 units per liter, reference range 8–52 U/l).

**Figure 5:**
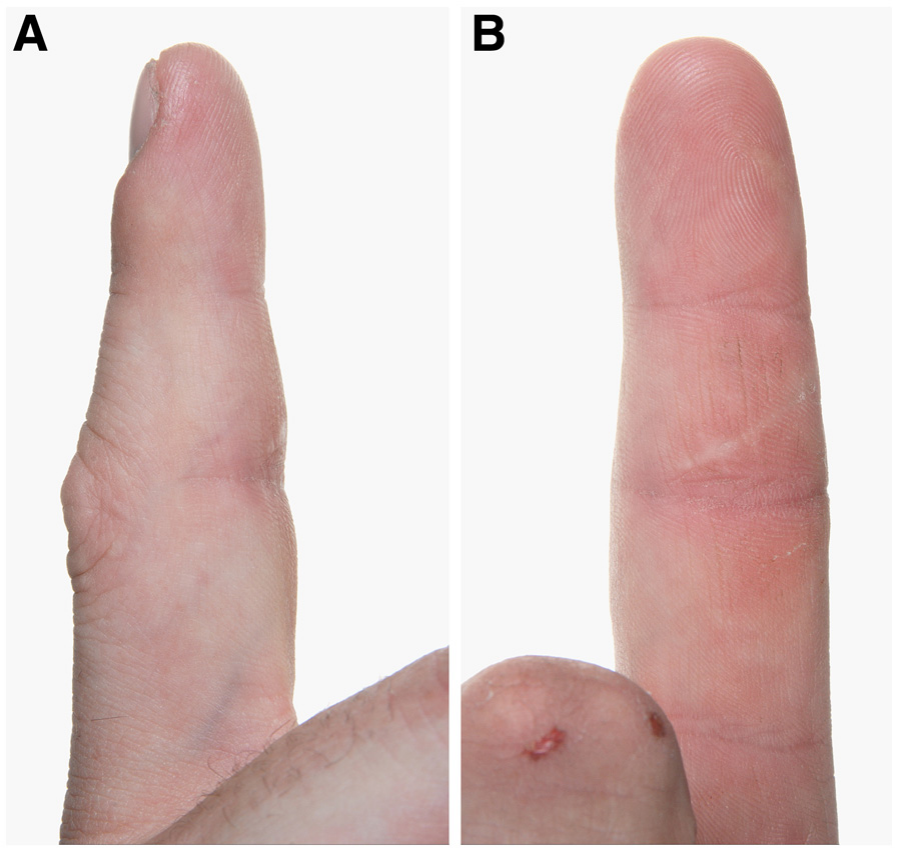
**A and B: lateral (A) and palmar (B) view of the right index finger one year after diagnosis.** After a one year treatment with corticosteroids only a slight swelling is left.

Oral corticosteroid therapy with an initial daily dose of 40 mg prednisone was started and led to an efficient diminution of the swelling of the right index finger. Hilar lymphadenopathy and lung infiltrates resolved. After 12 months therapy with a stepwise decrease of the corticosteroid dosage the swelling of the finger had almost vanished and function and range of motion was totally restored (Figure 5A and B). ACE-levels decreased to 26 U/l. At present, 36 months after first presentation, a maintenance dosage of 7,5 mg prednisone per day is needed as further reduction lead to a reactivation of sarcoidosis with increased ACE-levels (> 70 U/l).

## Discussion

Besides lymphadenopathy an involvement of lung or other organs is a feature of sarcoidosis. Even destruction and deformation of bones have so far been described [8]. In a recent case sarcoidosis emerged in a neuroma of the hand [9].

Scar sarcoidosis is a form of cutaneous sarcoidosis. It occurs after mechanical harms of the skin like cuts, but even a venous puncture or a tattoo seem to be sufficient to cause it [1,3,7,10]. In some cases scar infiltration is the only manifestation of the disease, more often the skin manifestation presents at the onset of a systemic disease [1]. One of the most important surgical differential diagnoses should be keloids [3]. In the described case no discoloration of the scar, no tenderness and no itchiness could be observed. Due to the MRI-scans (slight enhancement of contrast agent at the lesion´s border, Figure 2A and B) and the clinical aspect with a rapidly growing swelling a sarcoma (e.g. synovial sarcoma) or a giant cell tumour of the tendon sheath are important differential diagnoses. In general scar sarcoidosis involves different skin levels. In most of the cases an involvement of the superficial and deep dermis is described [5]. In our case a deep cut with injury of the deep flexor tendon led to a severe subcutaneous scar sarcoidosis. In case of scar sarcoidosis a radiological examination of the lungs as a work-up for systemic involvement should be performed [6]. It is known that scar sarcoidosis often resolves slowly and spontaneously, in other cases a topical appliance of hydroxychlorquin is effective [1,6]. Due to the massive local finding and proof of systemic extent of the sarcoidosis in the presented case a systemic therapy with corticosteroids for 12 month was recommended. This therapy led to fast and almost complete regression of the swelling, the finger´s function and range of motion was fully restored (Figure 5A and B). In addition, hilar lymphadenopathy resolved and pulmonary function improved during the period of corticosteroid therapy. 36 months after diagnosis a maintenance dosage of prednisone is needed to avoid reactivation of systemic sarcoidosis. Further surgical procedures were unnecessary.

## Conclusion

Every suspicious or progressive swelling needs a complete work-up with consideration of differential diagnoses and radiological examinations (especially plain radiographs and MRI scans with application of contrast agent). In case of cutaneous or subcutaneous swelling and a discoloration in the area of an old scar beside keloid a scar sarcoidosis is a possible differential diagnosis. For histological proof and exclusion of a malignant process a biopsy is to be performed. The biopsy offers the right diagnosis and thus leads to an appropriate, reasonable and successful treatment of sarcoidosis. In most cases satisfactory clinical results can be achieved by systemic or topic application of corticosteroids. Sometimes a long term therapy is needed, even if the local manifestation seems to be gone. Further local surgical procedures are not indicated.

## Consent

Written informed consent was obtained from the patient for publication of this case report and any accompanying images. A copy of the written consent is available for review by the Editor-in-Chief of this journal.

## Competing interests

The authors declare that they have no competing interests.

## Authors’ contribution

MH and JH are responsible for conception and writing of the manuscript, AS and GG have been involved in drafting and revising the manuscript. CS and CD were responsible for acquisition and analysis of data and findings. All authors read and approved the final manuscript.

## References

[B1] Marchell RM Judson MA Chronic cutaneous lesions of sarcoidosis Clin Dermatol 2007 25 3 295 302 10.1016/j.clindermatol.2007.03.007 17560307

[B2] Singal A Thami GP Goraya JS Scar sarcoidosis in childhood: case report and review of the literature Clin Exp Dermatol 2005 30 3 244 246 10.1111/j.1365-2230.2005.01727.x 15807680

[B3] Fernandez-Faith E McDonnell J Cutaneous sarcoidosis: differential diagnosis Clin Dermatol 2007 25 3 276 287 10.1016/j.clindermatol.2007.03.004 17560305

[B4] Yanardag H Pamuk ON Karayel T Cutaneous involvement in sarcoidosis: analysis of the features in 170 patients Respir Med 2003 97 8 978 982 10.1016/S0954-6111(03)00127-6 12924527

[B5] Mangas C Fernandez-Figueras MT Fite E Fernandez-Chico N Sabat M Ferrandiz C Clinical spectrum and histological analysis of 32 cases of specific cutaneous sarcoidosis J Cutan Pathol 2006 33 12 772 777 10.1111/j.1600-0560.2006.00563.x 17177936

[B6] Hong YC Na DJ Han SH Lee YD Cho YS Han MS A case of scar sarcoidosis Korean J Intern Med 2008 23 4 213 215 10.3904/kjim.2008.23.4.213 19119259 PMC2687677

[B7] Selim A Ehrsam E Atassi MB Khachemoune A Scar sarcoidosis: a case report and brief review Cutis 2006 78 6 418 422 17243430

[B8] Duckworth AD Hill AT Beggs I Patton JT Salter DM Porter DE Sarcoidosis presenting as a proximal phalangeal bony swelling-a case and review of the literature Hand (N Y) 2009 5 1 90 4 19517195 10.1007/s11552-009-9207-6 PMC2820614

[B9] Lee JH Jin W Kim GY A case of scar sarcoidosis presenting with a nodular mass infiltrating a neuroma in the hand J Hand Surg Eur Vol 2011 36 1 73 74 10.1177/1753193410381840 21169303

[B10] Rech G Balestri R Bardazzi F Piraccini BM Patrizi A Scar reactivation and dry cough Cleve Clin J Med 2011 78 6 375 376 10.3949/ccjm.78a.10132 21632907

